# Re-evaluating the Safety of Zolpidem: Risks and Clinical Implications

**DOI:** 10.5812/ijpr-164946

**Published:** 2025-10-15

**Authors:** Mohammad Babaeian, Ladan Ayazkhoo

**Affiliations:** 1 School of Pharmacy, Shahid Beheshti University of Medical Sciences, Tehran, Iran; 2 Department of Clinical Pharmacy, School of Pharmacy, Shahid Beheshti University of Medical Sciences, Tehran, Iran

**Keywords:** Zolpidem Abuse, Drug-Facilitated Sexual Assault (DFSA), Sleep Behavior Disorders, Insomnia Treatment, Hypnotic Risks, Cognitive Effects, Pharmacovigilance

*Dear Editor,*

The use of zolpidem for insomnia was once considered a safer alternative to benzodiazepines. However, emerging data and clinical reports have raised serious concerns regarding its safety, abuse potential, and misuse in criminal acts. Globally, zolpidem remains one of the most widely prescribed hypnotics, with millions of prescriptions issued annually across Europe, North America, and Asia (1). As healthcare professionals, we must critically evaluate whether its continued availability, especially in outpatient settings, is justified. However, a growing body of evidence suggests otherwise. A growing body of evidence suggests that careful reconsideration is needed.

**Failure as a Safer Alternative to Benzodiazepines**

Zolpidem was introduced to replace benzodiazepines as a lower-risk hypnotic. However, comparative data indicate that it shares many of the same risks, including tolerance, dependence, withdrawal, and cognitive impairment (1). Its adverse neuropsychiatric profile, including hallucinations, confusion, and paradoxical reactions such as irritability and agitation, challenges its positioning as a safer option. Despite its selective binding to GABAA receptors, reports of dose escalation and dependency patterns similar to those of benzodiazepines have accumulated (1).

**Use in Drug-Facilitated Sexual Assault and Criminal Misuse**

One of the most alarming concerns is the role of zolpidem in drug-facilitated sexual assault (DFSA) cases. For instance, a forensic toxicology case report describes a 23-year-old woman who reported sexual assault six days after the event, with intense amnesia. Advanced LC-MS/MS testing detected zolpidem at extremely low concentrations in her blood, urine, and hair, demonstrating how the drug can facilitate covert incapacitation and impede investigation (2, 3). Although such cases are statistically uncommon, recent reviews suggest that they are increasingly recognized and reported (3). This misuse poses serious public health and legal challenges, warranting urgent attention.

**Serious Side Effects and Widespread Harms**

Zolpidem has been associated with numerous dangerous effects beyond misuse. Complex sleep behaviors, such as sleep driving, sleepwalking, and binge eating, occur without full consciousness and often result in harm. Large-scale pharmacovigilance studies estimate these events in approximately 5 - 10% of users, although rates vary (4). Furthermore, long-term use has been associated with cognitive decline and an elevated risk of dementia, particularly among the elderly (5). However, causality remains uncertain, and confounding factors such as insomnia severity and comorbidities must be considered in future studies. Population-level data show that in the United States alone, more than 10,000 emergency department visits per year are attributed to zolpidem intoxication, with women disproportionately affected due to their slower metabolism of the drug (4). It is also commonly involved in polysubstance overdose presentations (6).

**Conclusions**

Zolpidem offers limited advantages over safer alternatives available in outpatient settings. However, it may retain a role in the short-term treatment of severe insomnia under close supervision. The risks of dependence and complex behaviors outweigh its modest benefits in DFSA. Consequently, zolpidem should be strictly limited to short-term, closely monitored use in inpatient settings, such as rapid procedural sedation, analogous to the controlled use of midazolam. Its widespread outpatient distribution should be re-evaluated in light of the accumulating evidence.
